# Independence of Valence and Reward in Emotional Word Processing: Electrophysiological Evidence

**DOI:** 10.3389/fpsyg.2013.00168

**Published:** 2013-04-08

**Authors:** Laura Kaltwasser, Stephanie Ries, Werner Sommer, Robert T. Knight, Roel M. Willems

**Affiliations:** ^1^Biologische Psychologie, Institut für Psychologie, Mathematisch – Naturwissenschaftliche Fakultät II, Humboldt-Universität zu BerlinBerlin, Germany; ^2^The Helen Wills Neuroscience Institute, University of California BerkeleyBerkeley, CA, USA; ^3^Donders Institute for Brain, Cognition and Behaviour, Radboud University NijmegenNijmegen, Netherlands; ^4^Max Planck Institute for PsycholinguisticsNijmegen, Netherlands

**Keywords:** emotion, reward expectancy, word processing, event-related potentials

## Abstract

Both emotion and reward are primary modulators of cognition: emotional word content enhances word processing, and reward expectancy similarly amplifies cognitive processing from the perceptual up to the executive control level. Here, we investigate how these primary regulators of cognition interact. We studied how the anticipation of gain or loss modulates the neural time course (event-related potentials, ERPs) related to processing of emotional words. Participants performed a semantic categorization task on emotional and neutral words, which were preceded by a cue indicating that performance could lead to monetary gain or loss. Emotion-related and reward-related effects occurred in different time windows, did not interact statistically, and showed different topographies. This speaks for an independence of reward expectancy and the processing of emotional word content. Therefore, privileged processing given to emotionally valenced words seems immune to short-term modulation of reward. Models of language comprehension should be able to incorporate effects of reward and emotion on language processing, and the current study argues for an architecture in which reward and emotion do not share a common neurobiological mechanism.

## Introduction

Emotional stimuli are special: they are processed faster and in a more elaborate manner than neutral stimuli. For instance, emotional facial expressions lead to faster reaction times (RTs) and larger event-related potentials (ERPs), compared to neutral faces (Eimer et al., 2003; Schacht and Sommer, 2009a). Similarly, emotional words (“murder,” “love”) elicit markedly different brain responses than more neutral words (for review, see Citron, 2012). In this study we examined whether the privileged processing of emotional words is influenced by another potent and primary regulator of cognition, namely the expectancy of reward. Recent work (for review, see Pessoa and Engelmann, 2010) shows that the expectation of reward or loss modulates cognitive processing such as spatial attention (Hickey et al., 2010), or visual working memory (Krawczyk et al., 2007). Here, we investigated whether emotional word processing is open to modulation by reward expectancy, or whether it is independent of such motivational effects. Due to its high temporal resolution, electroencephalography (EEG) allows for determining the influence of such modulations in different stages of word processing. The study reasonably complements the emerging research on extra-linguistic effects on the time course of language comprehension.

Emotional word content can modify several stages of word processing, from access to word meaning, to contextual integration, evaluation, and memory encoding (for review, see Kissler et al., 2006; Citron, 2012). Other studies report even earlier emotionality-dependent modulations of ERPs before or around 200 ms with particular task designs such as near-subliminal (Begleiter and Platz, 1969; Bernat et al., 2001; Ortigue et al., 2004) or hemifield presentation of words (Schapkin et al., 2000; Landis, 2006; Kanske and Kotz, 2007). Across input modalities (words, pictures, videos) emotional stimuli have shown a processing advantage over neutral ones (Eviatar and Zaidel, 1991; Dijksterhuis and Aarts, 2003), characterized at the neural level with amplified cortical responses (Cahill et al., 1996; Hamann et al., 1999; Herbert et al., 2009; Hofmann et al., 2009; Schacht and Sommer, 2009a,b), which is explained by the intrinsic relevance and salience of emotional stimuli (Lang et al., 1997).

Reward can enhance perceptual and executive control processes to achieve more efficient goal-directed behavior (Pessoa and Engelmann, 2010). For instance, reward expectancy can lead to top-down influences from prefrontal regions onto sensory areas involved in the process under study (Krugel et al., 2009; Philiastides et al., 2010). Most of the studies investigating the effect of reward expectancy on cognitive processes used fMRI. Thus the reported reward-induced cognitive enhancement was inferred from an increased blood-oxygen-level-dependent (BOLD) signal in task-relevant and value-related regions of the brain (Elliott et al., 2000; Breiter et al., 2001; Knutson et al., 2001; Delgado et al., 2003; Krugel et al., 2009; Pessoa and Engelmann, 2010; Philiastides et al., 2010). In electrophysiological research, the effect of reward on cognition is mainly studied in the context of stimulus-preceding (as in the SPN: for review, see van Boxtel and Böcker, 2004), error-related (as in the ERN: e.g., Holroyd et al., 2003; Nieuwenhuis et al., 2004) or feedback-related components (as in the FRN: e.g., Cohen et al., 2007; Hajcak et al., 2007; Bellebaum and Daum, 2008). However there are only few studies investigating the effect of reward cues on ERP components of perceptual and control processes in cognitive tasks offering monetary gain, as in Hickey et al. (2010) who found an increased P1 amplitude with reward-associated targets in a spatial attention task.

The present study tests for the presence of a common mechanism underlying emotion processing and reward expectancy by manipulating the two factors within the same experiment. A candidate common neurobiological mechanism is the dopamine system, given its involvement in reward (Elliott et al., 2000; Breiter et al., 2001; Knutson et al., 2001; Delgado et al., 2003), as well as emotional language (Schroeder et al., 2006, 2010; Moebes et al., 2008). Only few experiments in the language domain have hypothesized on how extra-linguistic factors may influence language processing, particularly how reward influences language comprehension, more specifically its temporal dynamics. For example in a recent study by Schacht et al. (2012) participants learned to associate previously unknown Chinese words with monetary gain, loss, or neither. When they were later required to distinguish the learned stimuli from novel distracters, an enhanced early (around 150 ms) as well as a later (550–700 ms) emotion effect could be measured for stimuli associated with monetary gain. This indicates that emotion effects in ERPs may arise in the absence of semantic meaning. But may the presence of monetary reward also alter emotion effects linked to semantic meaning?

In the present study we measured EEG from healthy participants while they performed a semantic categorization task (abstract – concrete) on words of different valence (positive, negative, neutral). Importantly, performance on each trial had a direct consequence for the participant: they could either win money, lose money, or neither (expected gain, expected loss, zero outcome expectancy). The presence or absence of gain was signaled by a cue presented 1 s before the word. Our working hypothesis was that if emotional valence and reward expectation interact via a common mechanism, we should observe interaction effects in behavioral measures and specific ERP components described below. On the contrary, if valence is shielded from the influence of reward, the difference between processing emotional and neutral words should be similar, irrespective of whether participants expected to win or lose on a given trial. As mentioned above, due to their connection to biologically significant system states, emotional words might induce a privileged allocation of neural resources, which is immune against the influence of short-term reward information.

We operationalized the emotional factor over the whole valence spectrum (e.g., positive, negative, neutral), instead of simply comparing one valence condition with neutral stimuli. This allowed to investigate whether ERPs are modulated by specific appetitive or defensive reactions (positive or negative emotional content), or whether the emotional nature of emotional words as such, irrespective of whether they are positive or negative, leads to an amplification of ERPs. Importantly, the present study was not designed to disentangle the effects of valence from the effects of arousal, as both differed between the emotional and neutral words (see Table 1).

**Table 1:** **Characteristics of the word stimuli used in the experiment**.

Variable	Positive	Neutral	Negative
Valence	7.53 (0.43)	5.24 (0.52)	2.42 (0.38)
Arousal	5.56 (0.91)	4.68 (0.82)	5.64 (0.84)
Concreteness	3.14 (1.48)	3.29 (1.48)	3.17 (1.36)
Word length (letters)	6.56 (2.03)	6.27 (1.85)	6.51 (2.26)
Word frequency (per million)	17.31 (27.46)	16.39 (28.83)	18.26 (35.96)

*For valence and arousal, ratings ranged from 1 (extremely negative valence or extremely low arousal) to 9 (extremely positive valence or extremely high arousal). For concreteness, ratings ranged from 1 (very abstract) to 5 (very concrete). Word frequency is based on counts for written English from the SUBTLEXus database. The emotional word categories were matched for concreteness, word length, and lexical frequency. Table shows the mean and standard deviation (in brackets)*.

Event-related potentials components of interest were the P2, early posterior negativity (EPN; e.g., Junghofer et al., 2001; Schupp et al., 2003), N400 and the late positive complex (LPC; e.g., Cuthbert et al., 2000; Schupp et al., 2000). The P2 is a distinct positive peak at anterior and central electrode sites around 150–250 ms, which was reported to be amplified by emotional content (Schapkin et al., 2000; Bernat et al., 2001; Herbert et al., 2006). However the P2 is not considered to be a typical emotion effect, but is rather associated with the selection of task-relevant perceptual items and has been shown to be more pronounced to a variety of target stimuli compared to distractor items (Potts et al., 1998; Potts and Tucker, 2001; Potts, 2004).

The EPN is a negativity at temporo-occipital electrode sites around 200–320 ms, which increases in amplitude to emotional pictures, facial expressions and words (Schupp et al., 2003, 2004a,b; Schacht and Sommer, 2009a,b; Scott et al., 2009). The EPN is suggested to result from reflex-like visual attention to emotionally significant and hence intrinsically relevant stimuli, which facilitates sensory encoding processes (Junghofer et al., 2001; Potts and Tucker, 2001; Schupp et al., 2004a; Schacht and Sommer, 2009a,b). The N400, a centro-parietal negativity arising around 400 ms after stimulus onset, has traditionally been considered as an index of semantic processing (Kutas and Federmeier, 2000; Hagoort et al., 2009). Some studies found a modulation of the N400 component by the emotional meaning of the eliciting words (Kissler et al., 2006; Kanske and Kotz, 2007; Citron, 2012). In addition, the modulation of the N400 by semantic expectancy changed across mood states of different valence (Federmeier et al., 2001). Importantly for our particular task design, the N400 has been shown enlarged in concrete compared to abstract words due to mechanisms of semantic integration (Kounios and Holcomb, 1994; Holcomb et al., 1999; Kanske and Kotz, 2007).

The LPC (also called the “late positive potential”) consists of an increased parietal positivity starting in the time range of the P300 component (around 300 ms) or later (Hajcak et al., 2010), lasting for several hundred milliseconds and presumably reflecting the more elaborate processing of emotional stimuli due to their motivational significance (Schupp et al., 2000). Various studies reported augmented LPC amplitudes for emotional pictures, facial expressions, and words of both positive and negative valence, as compared with neutral ones (Cuthbert et al., 2000; Schupp et al., 2000, 2003, 2004a,b; Fischler and Bradley, 2006; Schacht and Sommer, 2009a,b). These studies indicate that emotional influences on the LPC relate to the emotional intensity of stimuli rather than on low-level modality-dependent perceptual processes. This is consistent with the notion that this effect is being driven by motivational salience (for an excellent review, see Hajcak et al., 2010). Even though the LPC has been shown to be task-dependent (Fischler and Bradley, 2006; Schacht and Sommer, 2009b) it is a reliable indicator of emotionality in words (Kissler et al., 2006; Hajcak et al., 2010; Citron, 2012).

In order to examine the interaction between reward expectancy and emotional valence as specified above, we used a semantic categorization task on positive, negative, and neutral words, which were preceded by a cue signaling possible gain, loss, or zero outcome. The semantic categorization task was a concreteness decision, in order to ensure rather deep semantic processing without explicitly involving emotion as a task-relevant feature (Fischler and Bradley, 2006).

In accordance with previous studies, we expected emotional words to elicit larger amplitudes of the EPN and LPC components (as the most consistent emotion-dependent components) as well as faster RTs compared to neutral words. Furthermore, we assumed that the expectancy of gain and loss would lead to the allocation of additional neural resources as reflected in the enhancement of attention-related components such as the EPN or the P2 and improved behavioral performance compared to the zero outcome condition.

Most importantly, if emotional valence processing and reward expectation are dependent on the same mechanism, we should observe interaction effects in behavioral measures and specific ERP components. More specifically, one would expect an amplified emotion effect in the mean amplitude of emotion-related ERP components (EPN, LPC) in the expected gain and loss condition compared to the zero outcome condition due to enhanced cortical processing triggered by the expectancy of reward or avoidance of punishment.

Finally, as a side hypothesis linked to the semantic categorization task of concrete and abstract nouns and in line with the existing literature, we expected concreteness to elicit an enhanced N400 due to mechanisms of semantic integration.

## Materials and Methods

### Participants

Twenty-four UC Berkeley students (11 males; mean age = 21.2, range = 18–30), with English as their native language participated in the study for course credit or payment (US $10/h). Participants were right-handed (Oldfield, 1971), had normal or corrected-to-normal vision, and were without any neurological or neuropsychological disorder according to self-report. Prior to testing, each participant signed informed consent, and the study was approved by the university’s Institutional Review Board.

### Stimuli

Words were selected from the database of Affective Norms for English Words (ANEW) (Bradley and Lang, 2010). This database provides a set of normative emotional ratings (valence, arousal, dominance) based on the Self-Assessment Manikin (SAM) affective rating system on a 9-point scale (Bradley and Lang, 1994). Because we chose to use a concreteness judgment task in the main experiment, we asked 42 student volunteers (18 males) from UC Berkeley to evaluate the concreteness of 1760 positive, negative, and neutral words from the ANEW database on a 5-point scale (very abstract – rather abstract – neither/nor – rather concrete – very concrete). Words with a mean concreteness rating below 2.2 were considered abstract and above 3.8 as concrete.

The three valence categories (positive, negative, neutral) were matched in terms of word frequency, length, and concreteness using the software MATCH (van Casteren and Davis, 2007) (for statistics, see Table 1). Word frequency was taken from the SUBTLEXus database (Brysbaert and New, 2009). Because in the English language nouns can also be used as verbs, we excluded such ambiguities on the basis of the Common Part Of Speech (CPOS) measurement of the Medical Research Council Psycholinguistic Database of English Words (Coltheart, 1981).

The final word sample consisted of 300 English nouns of which one third was positive (valence rating ≥7), one third was negative (valence rating ≤3), and one third was neutral (valence rating between 4 and 6). Half the words in each valence category were concrete, the other was abstract.

Analysis of variance (ANOVA) of the final set of words yielded significant effects for valence rating [*F*(2,297) = 3297.77, *p* < 0.001] and arousal rating [*F*(2,297) = 39.06, *p* < 0.001] but not for word frequency, word length, and concreteness rating (all *F* < 1). A Pearson Chi-square test did not detect a significance difference in the frequency of noun type between the different emotional categories [χ^2^ (2, *N* = 300) = 1.09, *p* = 0.60]. As expected, *post hoc* comparisons (Games–Howell corrected for valence and concreteness rating since no equal variance could be assumed and Bonferroni corrected for all other factors) showed significant differences in valence between positive, negative, and neutral word groups and in arousal between the two emotional groups compared to the neutral word group (*p* < 0.001 for all). All other comparisons were not significant (*p* > 0.54). In summary, the three valence categories differed in terms of valence and arousal – as intended – but the other variables were controlled for.

### Task and procedure

**Figure 1:**
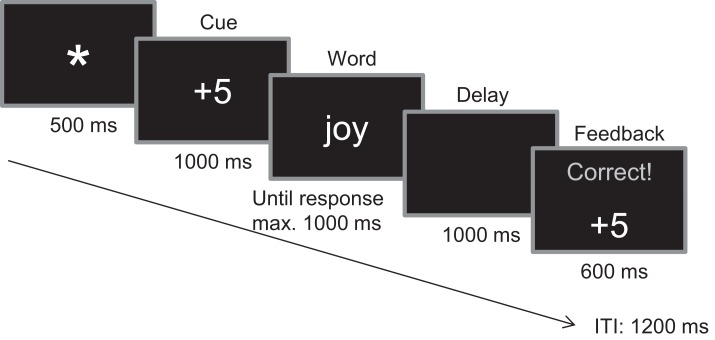
**Trial scheme**.

Each trial started with the presentation of a fixation star for 500 ms. Subsequently the participants saw one of three cue types (−5/0/+5 cents) for 1 s followed by a positive, negative, or neutral word. The task for the participants was to decide whether the word was abstract or concrete by pressing the left or right button of a five-Button SR Box. Participants were instructed that following a cue of “+5 cents” correct responses were rewarded and following a cue of “−5 cents” incorrect responses were punished (loss of 5 cents from current total). Assignment of keys to responses was counterbalanced across participants. The word stayed on the screen until the response occurred or until 1 s had elapsed. Responses were followed by 1 s black screen and a feedback screen for 600 ms which indicated the correctness of the answer, the monetary gain or loss in the current trial and the balance up to this point. Inter-trial-interval (ITI) was 1200 ms. Figure 1 depicts a sample trial scheme in the gain expectancy condition with a positively valenced word.

The experiment comprised 900 trials divided into nine blocks, which were run after a practice block with 18 trials. As each word was presented three times, it occurred once under each reward expectancy condition. The word repetition was blocked, that is, all words were shown before any word was repeated. The order in which the reward-word pairs were presented was mixed pseudo-randomly using the software MIX (van Casteren and Davis, 2006). There were never more than four identical responses and never more than three identical valence or reward expectancy conditions in a row. Since there are six different possible orders of the reward expectancy condition under which a word is presented, those orders were also counterbalanced across participants.

The experiment was run with E-prime software (E-prime 1.2 Professional, Pittsburgh, PA: Psychology Software Tools). The participants were seated in a dimly lit, sound-attenuated chamber facing a ViewSonic^®^ E90fb 19′′ monitor with a refresh rate of 70 Hz that was positioned 90 cm in front of them. Screen background was black throughout the entire experiment. The fixation star, the cue, and the word were presented in white within a visual angle of 2° of the participants’ field of view.

After the experiment participants were paid the money corresponding to their total balance ($11.63 on average, range: $6.90–$13.70) in addition to the per-hour-payment or course credit.

### EEG recording and processing

The EEG was recorded from 64 Ag/AgClActive-Two preamplified electrodes (BIOSEMI, Amsterdam; 10–20 system positions). The sampling rate was 1024 Hz (filters: DC to 268 Hz, 3 dB/octave). The passive reference electrode was placed over the left mastoid. The vertical EOG was recorded by means of an active surface electrode (Ag/AgCl) below the right eye. The horizontal EOG was recorded with two active surface electrodes positioned over the two outer canthi.

Unless stated otherwise all subsequent analyses of the EEG data were done with the Brain Vision Analyzer software (version 2.01; Brain Products GmbH, Munich, Germany). Data were re-referenced to the average of all electrodes (“average reference”), and data were band-pass filtered from 0.027 to 30 Hz (12 dB/octave, time constant for high pass filter: 6 s). Blink artifacts were corrected using the Multiple Source Eye Correction (MSEC) method (Berg and Scherg, 1994) with the software Brain Electrical Source Analysis (BESA 5.1, MEGIS Software GmbH, Munich, Germany). Subsequently, the continuous EEG data were segmented into intervals of 2200 ms, starting 200 ms prior to cue onset. Remaining artifacts were eliminated using automatic artifact rejection (maximal allowed voltage step: 15 μV/ms, maximal allowed difference of values in intervals: 200 μV, maximal/minimal allowed amplitude: ±150 μV, lowest allowed activity in intervals: 0.5 μV). Data from four participants were excluded from further analysis due to a rejection rate above 20%. The mean rejection rate of the remaining participants was 6.38% (1.33 – 16.13%). The rejection rate did not differ across conditions [*F*s(2,34) < 1, *p*s > 0.57]. ERPs from trials with correct responses were averaged for each valence category (positive, negative, neutral), reward cue (gain, loss, zero outcome), and semantic category (abstract, concrete). One more participant was excluded from further analysis because less than thirty trials remained in one of the sub-conditions of the 3 × 3 × 2 design, and another one because of non-compliance with instructions. The final sample consisted of 18 participants (nine males). For each condition the 200-ms before the onset of the cue were used as a baseline.

### Data analysis

For behavioral data the mean RTs for correct responses and the percentage of correct responses (accuracy) were analyzed with repeated measures ANOVAs involving the factors valence (positive, negative, neutral), reward cue (expected gain, expected loss, zero outcome expectancy), and concreteness (abstract, concrete). In case of significant main effects or interactions pairwise comparisons were conducted, and the resulting *p*-values were adjusted for multiple comparisons using the Bonferroni correction.

In order to assess experimental effects in ERP data, repeated measures ANOVAs were performed on the mean amplitude within consecutive time segments of 100 ms in the epochs described above (1 s before and after word onset). In the cue interval (1 s before word onset) the ANOVAs included the factors electrode (64 levels) and reward cue (three levels), whereas in the word interval the ANOVAs included the factors electrode (64 levels), valence (three levels), reward cue (three levels), and semantic category (two levels). By definition, the average reference sets the mean value of the ERP amplitude to zero across all electrodes within a given condition. Therefore, wherever all electrodes enter the ANOVA, only effects in interaction with electrodes are meaningful.

This exploratory procedure of 100 ms segments was chosen because previous findings reported a large temporal variance of emotion effects (Kissler et al., 2006; Schacht and Sommer, 2009b; Citron, 2012) and relatively little is known about the timing and spatial distribution of reward expectancy effects. As mentioned in the introduction, both EPN and LPC components extend over a wide time range. Therefore, our approach aimed to detect all possible emotion and reward expectancy effects present in the ERPs without *a priori* defined specific time windows or electrode locations.

Additionally, we determined the peak amplitude of the P2 in response to the cue and to the word. This was done with the Peak Detection Algorithm in the Brain Vision Analyzer software during the 150–250-ms interval after both cue and word on the electrodes AF3, AF4, AF7, AFz, C1, C2, CP1, CP2, CPz, Cz, F1, F2, F3, F4, F5, F7, FC1, FC2, FC3, FCz, FP1, FP2, FPz, and Fz. These electrodes were selected by visual inspection of the grand mean and the averages per subject. The mean P2 amplitudes (baseline-to-peak) were subjected to repeated measures ANOVAs with the factors electrode (24 levels), valence (three levels), reward cue (three levels), and semantic category (two levels).

For all ANOVAs on ERP data, Huynh–Feldt correction was applied in case of a violation of the assumption of sphericity, and the original degrees of freedom are reported. In all cases, for *post hoc* pairwise comparisons (*t*-tests) alpha levels were Bonferroni corrected.

## Results

### Behavioral results

**Figure 2:**
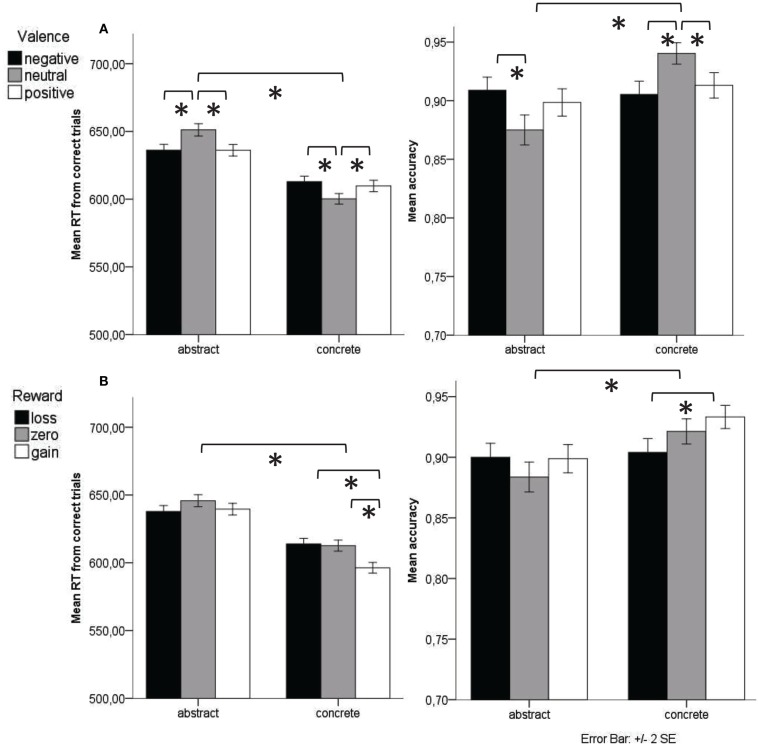
**Mean reaction time from correct trials and accuracy for valence (A) and reward expectancy (B) conditions in abstract and concrete words**.

Behavioral results are presented in Figure 2. Of all words 90.68% (SD = 4.07) were correctly categorized. RTs were significantly shorter for concrete (*M* = 608.21 ms, SD = 39.62) than for abstract (*M* = 642.06 ms, SD = 50.85) words [*F*(1,17) = 42.32, *p* < 0.001, $\eta_{p}^{2}=0.713$], and for words with expected gains (*M* = 618.36 ms, SD = 44.75) compared to expected losses (*M* = 626.87 ms, SD = 44.58) or words with zero outcome expectancy (*M* = 629.52 ms, SD = 44.29) [*F*(2,34) = 9.19, *p =* 0.001, $\eta_{p}^{2}=0.351$]. Valence had opposite effects for concrete and abstract words [interaction Valence × Concreteness: *F*(2,34) = 21.86, *p* < 0.001, $\eta_{p}^{2}=0.563$]. For both abstract and concrete words, neutral valence differed significantly from positive and negative words (abstract: both *p <* 0.001; concrete: *p* = 0.025 for neutral vs. positive and *p =* 0.001 for neutral vs. negative). However within abstract words, neutral words (*M* = 652.37 ms, SD = 49.52) slowed down RT compared to emotional words (positive: *M* = 636.80 ms, SD = 54.18; negative: *M* = 637.22 ms, SD = 50.17), while in concrete words neutral words (*M* = 601.13 ms, SD = 39.19) speeded up RT compared to emotional words (positive: *M* = 610.50 ms, SD = 41.60; negative: *M* = 613.76 ms, SD = 40.02) (see Figure 2A).

An interaction between reward expectancy and concreteness also reached significance [*F*(2,34) = 7.25, *p* = 0.002, $\eta_{p}^{2}=0.299$]. The difference between words with expected gain vs. expected loss and words with zero outcome expectancy was only significant for concrete (*p =* 0.004 and *p* = 0.011 respectively) but not for abstract words (*p* = 1.0 and *p* = 0.24 respectively). All other comparisons were not significant (*p* > 0.24, except for the difference between the zero outcome and loss condition within abstract words: *p* = 0.08).

The accuracy data are in line with the RT data. Again, ANOVAs on accuracy revealed significant main effects of reward expectancy [*F*(2,34) = 3.75, *p* = 0.034, $\eta_{p}^{2}=0.181$] and concreteness [*F*(1,17) = 10.93, *p* = 0.004, $\eta_{p}^{2}=0.391$]. Accuracy was higher for words with gain expectancy (*M* = 0.92, SD = 0.04) than with loss or zero outcome expectancy (*M* = 0.90, SD = 0.05 and *M* = 0.90, SD = 0.04, respectively); it was also higher for concrete than abstract words (*M* = 0.92, SD = 0.05 vs. *M* = 0.89, SD = 0.03). The factor valence did not reach significance (*F* < 1). As in the RT data, an interaction between valence and concreteness was significant [*F*(2,34) = 14.92, *p* < 0.001, $\eta_{p}^{2}=0.467$]. For concrete words, neutral valence differed significantly from positive and negative valence (*p* = 0.016 and *p* = 0.001). For abstract words, neutral valence differed only from negative valence (*p* = 0.012). Mirroring the RT results, in abstract words neutral valence (*M* = 0.875, SD = 0.07) led to lower accuracy than positive and negative valence (*M* = 0.898, SD = 0.05 and *M* = 0.909, SD = 0.04, respectively) whereas in concrete words neutral valence (*M* = 0.940, SD = 0.05) led to higher accuracy than positive or negative valence (*M* = 0.913, SD = 0.04 and *M* = 0.905, SD = 0.03, respectively). Finally reward expectancy interacted with concreteness [*F*(2,34) = 3.77, *p* = 0.033, $\eta_{p}^{2}=0.182$]. Pairwise comparison of this interaction within each semantic category showed that the only significant difference was within concrete words between expected gain and loss (*p* = 0.013). All other comparisons were not significant (*p* > 0.11).

In sum, the behavioral results did not show any interactions between valence and reward expectancy. Valence elicited opposite effects for concrete and abstract words: for abstract words, valence (positive, negative) made participants respond faster and more accurately as compared to neutral words, whereas for concrete words the opposite pattern was found (slower to emotional than to neutral words).

### EEG results

The statistical results for the omnibus ANOVAs in 100 ms segments with factors electrode, reward expectancy, valence, and concreteness are given in Table 2 for the cue and word intervals.

**Table 2:** **Results of the omnibus ANOVAs on mean amplitudes in the cue interval (top) and word interval (bottom) (ms after cue/word)**.

		0–100ms	100–200ms	200–300ms	300–400ms	400–500ms	500–600ms	600–700ms	700–800ms	800–900ms	900–1000ms
**CONDITION (IN CUE INTERVAL)**											
Reward × electrode (df = 126,2142)	*F*	0.753	**7.581**	**3.596**	**4.328**	**4.843**	1.978	1.471	1.508	1.439	1.526
	*p*	0.742	**0.000**	**0.001**	**0.000**	**0.000**	0.087	0.169	0.088	0.112	0.103
	$\eta_{p}^{2}$	0.042	**0.308**	**0.175**	**0.203**	**0.222**	0.104	0.080	0.081	0.078	0.082
	ε	0.131	**0.103**	**0.061**	**0.065**	**0.058**	0.041	0.067	0.138	0.143	0.111
**CONDITION (IN WORD INTERVAL)**											
Reward × electrode (df = 126,2142)	*F*	**2.229**	**2.284**	**2.935**	1.319	1.420	1.718	1.446	1.147	1.171	0.304
	*p*	**0.005**	**0.004**	**0.000**	0.219	0.153	0.070	0.128	0.310	0.276	0.737
	$\eta_{p}^{2}$	**0.116**	**0.118**	**0.147**	0.072	0.077	0.092	0.078	0.063	0.064	0.018
	ε	**0.128**	**0.124**	**0.116**	0.085	0.101	0.089	0.117	0.131	0.161	0.155
Valence × electrode (df = 126,2142)	*F*	1.597	1.386	1.286	1.335	**2.241**	**1.962**	**1.705**	1.196	0.892	1.217
	*p*	0.065	0.137	0.153	0.162	**0.007**	**0.019**	**0.040**	0.263	0.596	0.246
	$\eta_{p}^{2}$	0.086	0.075	0.070	0.073	**0.116**	**0.103**	**0.091**	0.066	0.050	0.067
	ε	0.134	0.142	0.221	0.147	**0.111**	**0.116**	**0.138**	0.141	0.157	0.142
Concreteness × electrode (df = 63,1071)	*F*	0.766	1.249	1.243	**7.905**	**2.690**	**6.249**	**3.284**	1.004	1.402	1.521
	*p*	0.623	0.262	0.277	**0.000**	**0.019**	**0.000**	**0.003**	0.422	0.211	0.165
	$\eta_{p}^{2}$	0.043	0.068	0.068	**0.317**	**0.137**	**0.269**	**0.162**	0.056	0.076	0.082
	ε	0.117	0.161	0.131	**0.138**	**0.094**	**0.089**	**0.116**	0.081	0.111	0.114
Reward × valence × electrode (df = 252,4284)	*F*	0.984	0.808	0.818	0.760	0.851	1.003	1.158	1.123	1.002	0.992
	*p*	0.490	0.736	0.719	0.821	0.724	0.466	0.250	0.351	0.465	0.479
	$\eta_{p}^{2}$	0.055	0.045	0.046	0.043	0.048	0.056	0.064	0.062	0.056	0.055
	ε	0.110	0.101	0.099	0.121	0.150	0.138	0.136	0.111	0.119	0.113
Valence × concreteness × electrode (df = 126,2142)	*F*	1.038	0.782	0.747	0.905	**2.571**	**3.289**	**1.891**	0.886	1.243	1.234
	*p*	0.416	0.759	0.817	0.614	**0.000**	**0.000**	**0.006**	0.619	0.205	0.215
	$\eta_{p}^{2}$	0.058	0.044	0.042	0.051	**0.131**	**0.162**	**0.100**	0.050	0.068	0.068
	ε	0.168	0.188	0.210	0.237	**0.185**	**0.235**	**0.204**	0.186	0.180	0.174
Reward × concreteness × electrode (df = 126,2142)	*F*	1.030	0.868	0.704	1.188	0.842	1.062	1.381	1.262	0.829	0.774
	*p*	0.423	0.601	0.826	0.244	0.684	0.384	0.100	0.180	0.692	0.756
	$\eta_{p}^{2}$	0.057	0.049	0.040	0.065	0.047	0.059	0.075	0.069	0.046	0.034
	ε	0.102	0.119	0.163	0.201	0.195	0.205	0.210	0.202	0.178	0.171

*Only condition × electrode interactions are reported (see text). The fourway interaction reward expectancy × valence × concreteness × electrode never reached significance (*p* > 0.1). df, original degrees of freedom; significant intervals in bold*.

#### Reward expectancy effects

**Figure 3:**
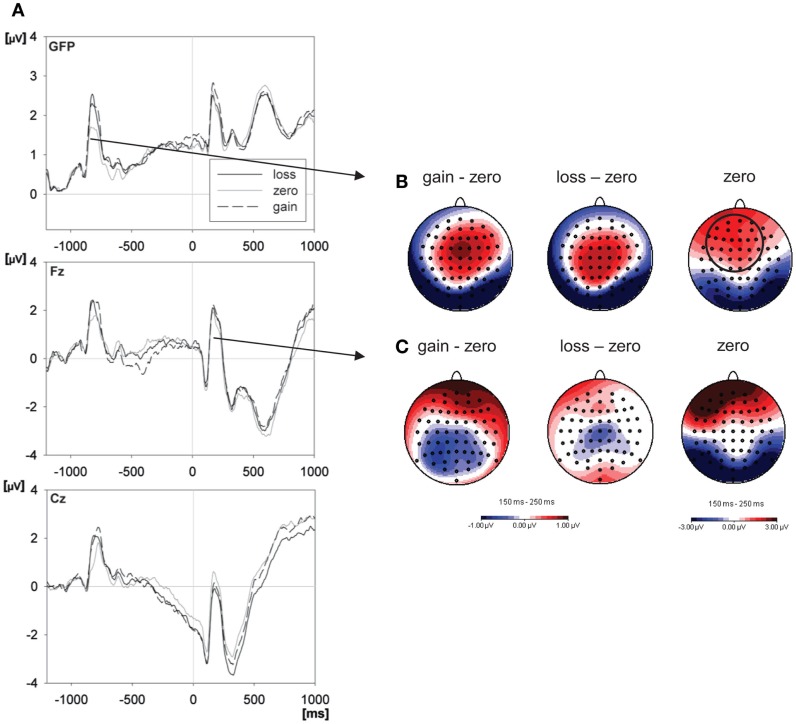
**Effects of reward expectancy on ERPs**. **(A)** The left panel depicts the grand mean ERP waveforms from global field power (GFP) – which reflects the overall ERP activity across the scalp at any given moment, frontal (Fz), and central (Cz) electrodes, within the gain, loss, and zero outcome expectancy condition. The word was presented at 0 ms, the cue at −1000 ms. The right panel shows the scalp distributions (standard amplitude subtraction) of the differences between expected gain and zero outcome condition and between expected loss and zero outcome condition and, further, the distribution of ERPs in the zero outcome condition within the 150–250-ms interval after the cue **(B)** and after the word **(C)** (electrodes of the P2 peak analysis are indicated by the circle).

Figure 3 shows grand average ERPs for the different reward expectancy conditions and topographies for the differences between these conditions. A reward expectancy effect after the cue was characterized by central positivities combined with frontal and occipital negativities (see Figure 3B), resembling the topography of the EPN (frontal positivity with occipital negativity). This was confirmed by a significant effect of the factor reward expectancy 100–500 ms after the cue. The reward expectancy effect in the cue interval consisted of larger fronto-central amplitudes for the gain [*F*(63,1071) = 6.85, *p* < 0.001, $\eta_{p}^{2}=0.287$, ε = 0.084] and loss [*F*(63,1071) = 5.30, *p* = 0.001, $\eta_{p}^{2}=0.238$, ε = 0.067] compared to the zero cue, but not between the gain and loss cue (*F* < 1) (see Figure 3A).

The reward expectancy effect in the word interval showed fronto-temporal positivities and centro-parietal negativities (see Figure 3C). This topography consisted of larger amplitudes for words with expected gain compared to both words with expected loss [*F*(63,1071) = 2.46, *p* = 0.015, $\eta_{p}^{2}=0.127$, ε = 0.132] and words with zero outcome cue [*F*(63,1071) = 3.55, *p* = 0.001, $\eta_{p}^{2}=0.173$, ε = 0.119] in the interval 0–300 ms after word onset (see Figure 3A). The other pairwise comparisons within the reward expectancy conditions did not reach significance (*p* > 0.41).

ANOVA of the P2 peak amplitude revealed a significant modulation by the factor reward expectancy in both the cue interval [*F*(46,782) = 2.51, *p* = 0.009, $\eta_{p}^{2}=0.128$, ε = 0.210] and the word interval [*F*(46,782) = 2.66, *p* < 0.001, $\eta_{p}^{2}=0.135$, ε = 0.369]. In both intervals the P2 potential was significantly more pronounced for the gain expectancy relative to the zero outcome expectancy condition [P2 cue: *F*(23,391) = 4.59, *p* < 0.001, $\eta_{p}^{2}=0.213$, ε = 0.371; P2 word: *F*(23,391) = 3.66, *p* < 0.001, $\eta_{p}^{2}=0.177$, ε = 0.369] and in the word interval also relative to the loss expectancy condition [*F*(23,391) = 2.51, *p* = 0.013, $\eta_{p}^{2}=0.128$, ε = 0.361]. The other pairwise comparisons in the P2 analysis within the reward expectancy condition did not reach significance (*p* > 0.15).

#### Valence effect

**Figure 4:**
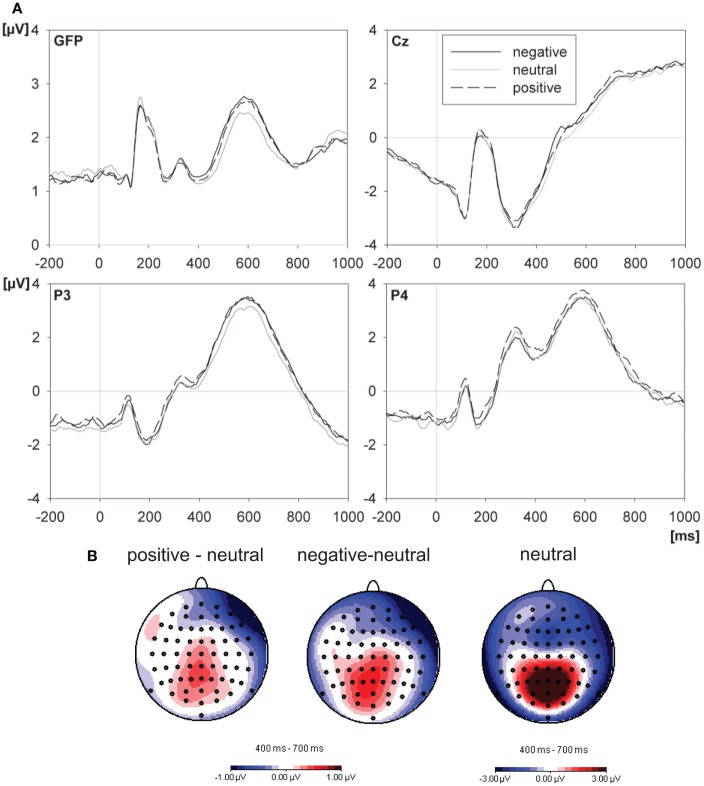
**Effects of emotional word valence on ERPs**. **(A)** The upper panel depicts the grand mean ERP waveforms from GFP, central (Cz), and parietal (P3 and P4) electrodes, elicited by emotionally positive, negative, and neutral words. The word was presented at 0 ms. **(B)** The lower panel shows the scalp distributions (standard amplitude subtraction) of the differences between positive and neutral words and between negative and neutral words and, further, the distribution of ERPs to neutral words within the 400–700-ms interval after the word.

Figure 4 shows grand average ERPs for the different valence conditions and topographies for the differences between these conditions. The valence effect was composed of enhanced positivities at centro-parietal sites for positive and negative words compared to neutral words. There was a main effect of emotional valence 400–700 ms after word onset which consisted of significant differences between neutral words and both positive [*F*(63,1071) = 2.31, *p* = 0.005, $\eta_{p}^{2}=0.120$, ε = 0.226] and negative [*F*(63,1071) = 2.90, *p* = 0.008, $\eta_{p}^{2}=0.146$, ε = 0.109] words (see Figure 4A and below). There was no significant difference between positive and negative words (*F* < 1).

In line with previous studies (Schacht and Sommer, 2009a,b), we performed additional region-of-interest analyses (ROI) for the emotion effects EPN and LPC:

For the EPN, regional analysis on activity of eight occipital electrodes (P9, PO7, O1, Oz, Iz, O2, PO8, P10) in the common time-interval of the EPN (200–400 ms) did not show a significant modulation by valence [*F*(2,34) = 0.11, *p* = 0.889, $\eta_{p}^{2}=0.006$, ε = 1] and no interaction between valence and reward expectancy [*F*(2,34) = 1.17, *p* = 0.331, $\eta_{p}^{2}=0.064$, ε = 0.893].

For the LPC, regional analysis on activity of ten centro-parietal electrodes (C3, C4, CPz, CP1, CP2, FC1, FC2, Pz, P3, P4) verified the late main effect of emotion [*F*(2,34) = 8.75, *p* = 0.001, $\eta_{p}^{2}=0.340$, ε = 0.992], comprising a significantly pronounced LPC amplitude for positive compared to neutral words [*F*(1,17) = 10.63, *p* = 0.005, $\eta_{p}^{2}=0.385$, ε = 1.0], as well as for negative compared to neutral words [*F*(1,17) = 12.85, *p* = 0.002, $\eta_{p}^{2}=0.431$, ε = 1.0], but not between positive and negative words (*F* < 1). Similarly, this ROI-Analysis did not reveal a significant interaction between valence and reward expectancy (*F* < 1).

**Figure 5:**
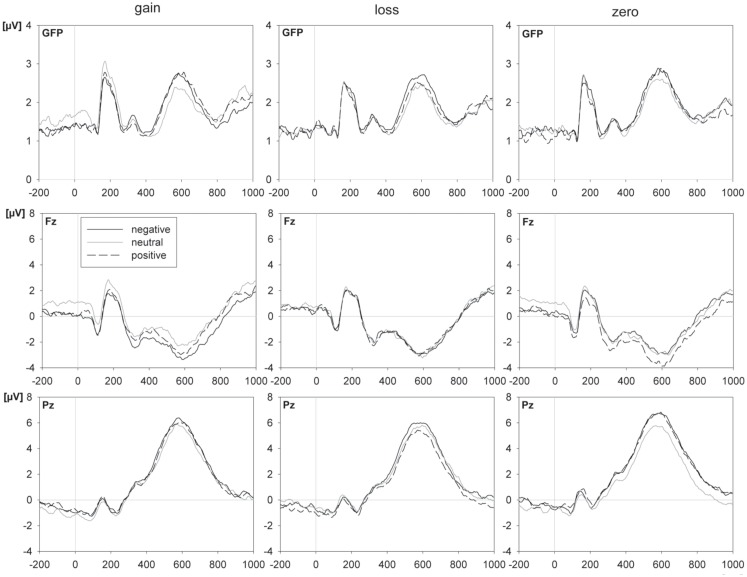
**ERP effects of emotional word valence as a function of reward expectancy**.

We did not find an interaction between the factors reward expectancy and valence neither in the omnibus ANOVA in 100 ms segments nor in the ROI analyses of EPN and LPC or in the analysis of the P2. Figure 5 shows grand average ERPs of emotional word valence as a function of reward expectancy.

#### Concreteness effect

**Figure 6:**
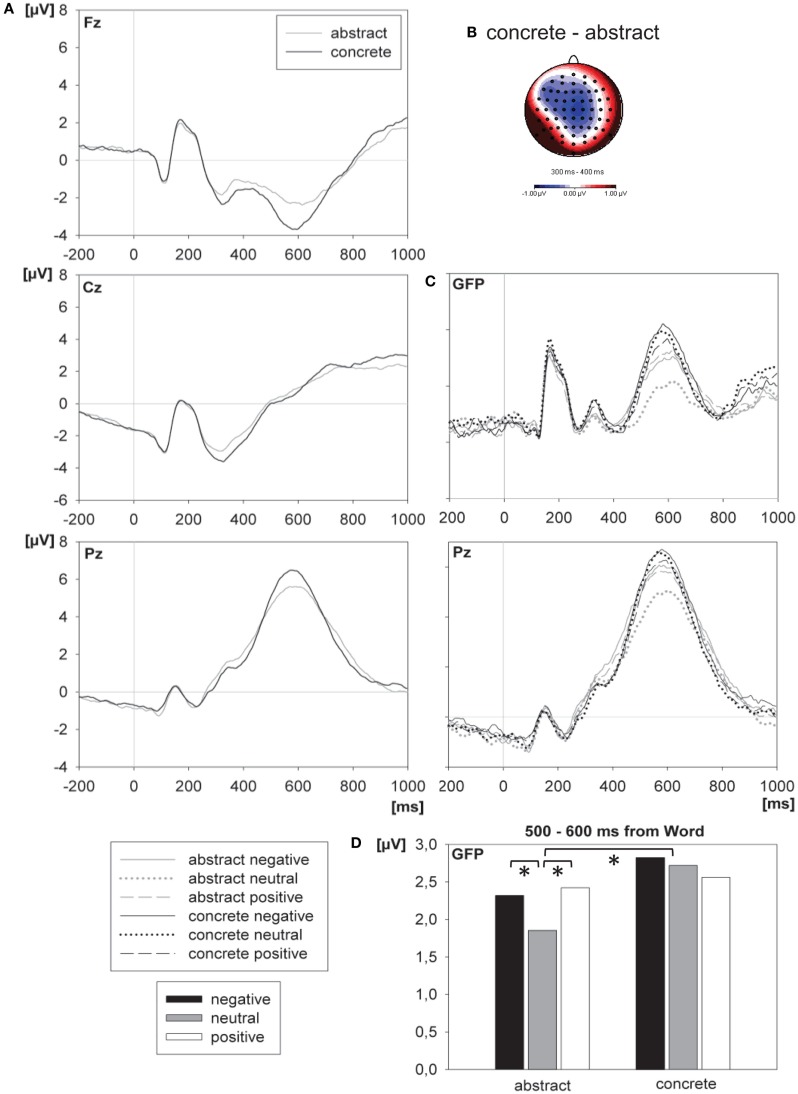
**Effects of concreteness and valence on ERPs**. **(A)** The panel depicts the grand mean ERP waveforms from frontal (Fz), central (Cz), and parietal (Pz) electrodes, elicited by abstract and concrete words. The word was presented at 0 ms. **(B)** The upper right panel shows the scalp distributions (standard amplitude subtraction) of the differences between concrete and abstract words within the 300–400-ms interval after the word. **(C)** The middle right panel depicts the grand mean ERP waveforms from GFP and parietal (Pz) electrode, elicited by abstract and concrete words in each valence condition. The word was presented at 0 ms. **(D)** The lower right panel shows the corresponding mean amplitude of the GFP within the 500–600-ms interval after the word. An asterisk marks a significant difference between conditions.

In accordance with previous studies (Kanske and Kotz, 2007) concrete words elicited a larger N400 than abstract words (see Figure 6A, Cz), which consisted of central negativities with a slight left lateralization at anterior sites (see Figure 6B). Concreteness had a significant effect on ERP data 300–700 ms after word onset.

The P2 component was not significantly affected by concreteness [*F*(23,391) = 1.03, *p* = 0.41, $\eta_{p}^{2}=0.057$, ε = 0.276].

#### Valence × concreteness interaction

Mirroring the behavioral results valence interacted significantly with concreteness in the 400–700-ms after the word. As can be seen in Figure 6C the valence effect was more pronounced in abstract words compared to concrete words. *Post hoc* tests revealed that the interaction consisted of significantly more pronounced parietal positivities for both abstract negative [*F*(63,1071) = 3.29, *p* = 0.002, $\eta_{p}^{2}=0.162$, ε = 0.129] and abstract positive [*F*(63,1071) = 4.85, *p* < 0.001, $\eta_{p}^{2}=0.222$, ε = 0.201] compared to abstract neutral words, as well as a significantly reduced parietal positivity for concrete negative compared to concrete neutral words [*F*(63,1071) = 1.93, *p* = 0.027, $\eta_{p}^{2}=0.102$, ε = 0.208]. All other pairwise comparisons did not reveal statistically significant differences between conditions (*p* > 0.25). Because of the similar pattern of the interaction effect in RT (see Figure 2) and in the mean amplitude of the GFP (see Figure 6D), we ran an additional ANOVA with the factors valence (three levels) and concreteness (two levels) to further assess the valence × concreteness interaction in the mean amplitude of the GFP, which reflects the overall ERP activity across the scalp at any given moment. Apart from a main effect of valence [*F*(2,34) = 5.50, *p* = 0.009, $\eta_{p}^{2}=0.244$] and concreteness [*F*(1,17) = 6.04, *p* = 0.025, $\eta_{p}^{2}=0.262$], this *post hoc* analysis revealed a significant difference between neutral abstract words compared to both classes of emotional abstract words (positive: *p* = 0.039; negative: *p* = 0.018), as well as a significant difference between abstract and concrete neutral words (*p* = 0.008), where neutral abstract words yielded smaller amplitudes than both emotional abstract words and concrete words. All other pairwise comparisons were non-significant (*p* > 0.1).

## Discussion

The aim of the present study was to assess the interplay of emotion, as reflected in word-inherent valence, and reward, as reflected in the expectancy of monetary gain, loss, or none of both. Our working hypothesis was that if emotional valence and reward expectation interact via a common mechanism, we should observe interaction effects in behavioral measures (RT or accuracy) and specific components of the ERP (P2, EPN, N400, LPC). Therefore, participants undertook a semantic decision task on emotional words which were preceded by cues indicating gain, loss, or neither.

While we found significant main effects of reward expectancy and word-inherent valence on ERPs in response to words, these factors did not interact at any time point in the present study. This finding was corroborated by the behavioral measures, which indicated a significant main effect of reward expectancy on RT and accuracy but also showed no interaction between reward expectancy and valence. We do not think that this null result concerning the interaction is due to a lack in experimental power because we observed significant main effects of each factor. Furthermore, Table 2 shows that the effect size $\left(\eta_{p}^{2}\right)$ of the interaction between reward expectancy and word valence is relatively small (around 0.05) compared to the effect size of the significant main effects (>0.1).

Instead, our results speak for independent mechanisms involved in the processing of reward expectancy and word-inherent valence. This claim is further supported by a dissimilarity of the timing and the topography of reward expectancy and valence effects in ERPs. Our findings regarding the influence of reward expectancy in language processing are therefore in contrast to studies who found an enhancement of cognitive and neurophysiological processes in other higher cognitive functions such as spatial attention, memory, and executive control (Hickey et al., 2010; Pessoa and Engelmann, 2010).

Interestingly, the semantic variable of concreteness, which was not linked to our working hypothesis but was an element of our experimental task, had a major influence on ERP and behavioral data: not only did concreteness exert a significant influence on both measures – it also interacted with valence (behavior and ERP) and reward expectancy (only behavior).

As outlined in the Section “Methods” our analysis procedure of the ERP data included an exploratory analysis of 100 ms segments in the cue- and word segment due to the large temporal invariance of emotion effects in previous studies and the sparse evidence on timing of reward expectancy effects. This first step was then confirmed by ROI analyses of emotion and reward expectancy related components of interest (P2, EPN, LPC). We therefore emphasize the exploratory character of our ERP analyses and results. We will now discuss the ERP effects related to reward expectancy, valence, and the valence × concreteness interaction in turn.

### Effects of emotional valence

Replicating previous studies, emotional words elicited larger LPC amplitudes than neutral words (Fischler and Bradley, 2006; Schacht and Sommer, 2009a,b). ROI analyses revealed that this late emotion effect was characterized by the typical latency (400–700 ms) and topography (parietal positivities) of the LPC (Hajcak et al., 2010). Moreover this finding is in line with studies on affective picture processing (Cuthbert et al., 2000; Schupp et al., 2000, 2003, 2004a) and emotional expressions in face recognition (Schupp et al., 2004b; Schacht and Sommer, 2009a). Importantly and as in most of the studies cited here, positive and negative words were quite similar in their neurophysiological profiles as compared to neutral words. This finding suggests that it might be the intrinsic relevance of emotional stimuli *per se* that is critical in modulating the ERPs during language processing, rather than a specific coding of appetitive (positive) vs. defensive (negative) reactions. As outlined in the introduction, the LPC in the present study can be interpreted as a valence-unspecific elaborative processing of intrinsically salient emotional stimuli. Emotionality allocates neural resources even though it is not related to task requirements or monetary gain.

We did not find an early emotion effect such as the EPN. This can be explained by task effects, which are known to have an important influence on early emotion effects (Schacht and Sommer, 2009b; Bayer et al., 2010; Rellecke et al., 2011). It seems plausible that particular task demands prevent the automatic or reflex-like attention capture by emotion as it is postulated for the EPN by Potts and Tucker (2001) and others (Junghofer et al., 2001; Schupp et al., 2004b; Schacht and Sommer, 2009a,b). If a semantic emotion-unrelated analysis is necessary, as in a semantic context decision (Bayer et al., 2010) or a concreteness decision like in the present study, task requirements demanding cognitive resources and competing with the involuntary attention capture by emotion might prevent the occurrence of an EPN. This is further corroborated by the only other study we know of that applied a concreteness decision to emotional words and also failed to find an early emotion effect such as the EPN (Naumann et al., 1997).

### Effects of reward expectancy

In line with the literature on reward expectancy cited in the introduction we expected higher amplitudes in task-related ERP components in situations of reward- or loss-expectancy, reflecting an increased allocation of neural resources to value-related stimuli compared to stimuli with zero outcome expectancy. Indeed, we found increased P2 amplitudes in response to both cue and word in the gain expectancy condition. Interestingly the P2 in the cue interval showed a topography similar to the EPN which did not differ between the gain and the loss condition. One interpretation could be that the gain vs. loss manipulation was not powerful enough. However this pattern is typical for the EPN which is attributed to processes of attention capture by emotionally salient events such as the announcement of reward or punishment (Potts and Tucker, 2001).

The P2 is associated with the selection of task-relevant perceptual items and has been shown to be more pronounced to a variety of target stimuli compared to distractor items, including auditory and visual, and in a variety of response tasks, including overt and covert responding, suggesting that it is not sensitive to specific perceptual features or response options but rather to the relevance of the item to the current task (Potts et al., 1998; Potts and Tucker, 2001; Potts, 2004). As mentioned in the introduction there are few electrophysiological studies (as in Hickey et al., 2010) which examine the effect of reward expectancy between the stimuli and the response. Hickey et al. found an earlier modulation of ERPs (P1) by reward expectancy than we did (P2). This can be explained by the fact that they used a spatial attention task with figural stimuli rather than a word processing paradigm as we did. We are therefore one of the first to show that the expectancy of reward as indicated by a cue modulates attention-related ERP components related to the processing of (another) stimuli (but, see Schacht et al., 2012).

### Interaction of valence and reward

The present study was designed to investigate the interplay of word-inherent valence and reward expectancy. Our finding of an independence and a successive timing of the two effects speaks for separate and sequential rather than parallel processes. Importantly, our results do not imply that emotion is generally separated from a reward or punishment system, but that emotional semantics are independent from a reward or punishment system coding for short-term monetary gain. The contextual valence created by the positive, negative, or neutral reward cue does not change the processing of subsequent emotional content in words, suggesting that emotional words are immune to the short-term influences of reward.

The lack of an interaction between word-inherent valence and reward expectancy may be explained by top-down processes triggered by reward expectancy and selective attention to concreteness, which might have hidden subtle effects of intrinsic word valence. However our results find support in an fMRI study by Wittmann et al. (2008), using affective picture stimuli. They report that reward-related processing in the ventral striatum was affected by the emotional valence of subsequently presented pictures but that emotional processing in the amygdala was immune to the expectation of reward. Several neuroimaging and lesion studies point out that the amygdala plays a crucial role in the privileged processing of emotional words (Isenberg et al., 1999; Anderson and Phelps, 2001; Garavan et al., 2001; Hamann and Mao, 2002; Naccache et al., 2005). Although using different brain imaging techniques, our results and the mentioned study from Wittmann et al. suggest that there are neural correlates of emotional semantics that are unaffected by the expectancy of monetary gain.

Lastly, we should address the ecological validity of our design. Our hypotheses were inspired by the similarity of the enhancement of cognitive and neurophysiological processes by reward expectancy and emotionality. As discussed in the introduction, one common ground could be the dopamine system which has been shown to be involved in the coding of reward (Alexander et al., 1986; Elliott et al., 2000; Breiter et al., 2001; Knutson et al., 2001; Delgado et al., 2003) as well as in emotional language processing (Schroeder et al., 2006, 2010; Moebes et al., 2008). However which real-world situations reflect an interaction between reward expectancy and emotional valence in word processing? One could for example think about a salary negotiation conversation which the employe expects to result in a raise, and then bad words come along; or an academic meeting where the student expects harsh words by his supervisor about his data analysis, while the supervisor only expresses kind words. Our results suggest that the intrinsic emotional valence of the expressed words will be unaffected by the prior reward expectancy. So, in the latter example, even though the student expected punishment by harsh words, the positive feedback by his supervisor will be processed as such.

### Interaction of valence and concreteness

In line with previous studies we found a concreteness effect, characterized at the behavioral level with faster and more accurate reactions and at the neurophysiological level with an enlarged N400 for concrete words relative to abstract words. This finding has been related to a greater activation of semantic neighbors by concrete words (Kounios and Holcomb, 1994; Kanske and Kotz, 2007). According to the context availability model, concrete words showed an advantage over abstract words due to denser associations to contextual knowledge (Schwanenflugel and Shoben, 1983; Schwanenflugel, 1991). As such, the difference in the N400 represents differences in the activation of the semantic network. Concrete words activate more semantic context and thus elicit an enlarged N400 (Holcomb et al., 1999).

Notably the interaction of valence and concreteness is characterized in behavioral and neurophysiological data by a similarity of emotional abstract words to concrete words. Emotionality in abstract words leads to a decrease of reaction time as well as an increase of accuracy, whereas emotionality in concrete words increases reaction time and decreases accuracy (see Figure 2). This pattern in the behavioral measure is corroborated by a recent study which examined whether lexical processing is sensitive to the dimension of emotional experience (i.e., the ease with which words evoke emotional experience) and found that in abstract nouns, emotional experience was associated with faster and more accurate categorization, whereas in concrete nouns, emotional experience was linked to less accurate categorization (Newcombe et al., 2012).

At the neuronal level the valence effect was more pronounced in abstract words than in concrete words (see Figures 6C,D). This is at odds with the results of Kanske and Kotz (2007) who found an enhanced LPC for concrete negative words compared to concrete positive and neutral words with no differences observed in abstract words. Since their data were obtained with a visual hemifield lexical decision task with concrete German nouns, it is possible that task effects are responsible for the differing results (also, see Palazova et al., in press). However, more ERP studies combining valence and concreteness in one design are necessary in order to draw conclusions.

Our results more likely support the idea that emotional abstract words have a higher imageability compared to neutral abstract words (Paivio, 1986). This is in line with a recent study stating that emotional content plays a crucial role in the processing and representation of abstract concepts (Kousta et al., 2011). Accordingly abstract words are linked to concreteness by means of emotion. This can be seen as in line with theories of embodied semantics (Vigliocco et al., 2004; Zwaan, 2004) which are based on the core assumption that the representation and processing of semantic information recruit the same neural systems that are engaged during perception and action. In abstract words, emotion is considered to be another type of experiential information playing an important role in learning, representing, and processing (Vigliocco et al., 2009). In this case the interaction between valence and concreteness might index a differential activation of mental imagery by abstract emotional and neutral words (but, see also Citron, 2012).

## Conclusion

In the present study, we investigated the effects of two major regulators of cognition, namely reward expectancy and emotion, on word processing. Reward expectancy is a primary modulator of cognition leading to improved behavioral performance and enhanced early ERP components that have previously been linked to attention processes (P2). Similarly, the emotional valence of words amplified cortical responses in a LPC which has been associated with the elaborate processing of biologically significant system states.

In the present study we did not find evidence for a common mechanism behind reward and emotion effects in word comprehension. Rather, emotion-related and reward-related effects occurred in different time windows in the course of word processing, did not interact, and showed different topographies. The fact that we found stable main effects of valence and reward expectancy which differed by means of timing and topography speaks against a lack of experimental power as the cause of the absence of an interaction. The results rather suggest that reward expectancy and valence, as operationalized in our study, were processed by different underlying cognitive systems. This speaks for an independence of reward expectancy and the processing of emotional content of a word. Emotional semantics might be processed in a privileged manner which is not affected by short-term information of monetary gain or loss. So while classical models of language comprehension should be extended to include the influence of factors such as reward and emotion, the current study argues for an architecture in which valence and reward do not operate through a similar mechanism on word processing.

## Conflict of Interest Statement

The authors declare that the research was conducted in the absence of any commercial or financial relationships that could be construed as a potential conflict of interest.
